# Repurposing of FDA-Approved NSAIDs for DPP-4 Inhibition as an Alternative for Diabetes Mellitus Treatment: Computational and in Vitro Study

**DOI:** 10.3390/pharmaceutics11050238

**Published:** 2019-05-17

**Authors:** Veera C. S. R. Chittepu, Poonam Kalhotra, Tzayhri Osorio-Gallardo, Tzayhri Gallardo-Velázquez, Guillermo Osorio-Revilla

**Affiliations:** 1 Departamento de Ingenieria Bioquimica, Escuela Nacional de Ciencias Biologicas, Instituto Politecnico Nacional, Av. Wilfrido Massieu S/N, Col. Unidad Profesional Adolfo Lopez Mateos, Zacatenco, C.P. Ciudad de Mexico 07738, Mexico; veerareddy9@gmail.com; 2 Departamento de Biofisica, Escuela Nacional de Ciencias Biologicas, Instituto Politecnico Nacional, Prolongacion de Carpio y Plan de Ayala S/N, Col. Santo Tomas, CP. Ciudad de Mexico 11340, Mexico; kalhotrapoonam@gmail.com (P.K.); gtzayhri@yahoo.com (T.G.-V.); 3 Departamento de Microbiologia e Immunologia, Facultad de Medicina Vetererneria Y Zootecnia, Universidad Nacional Autonoma de Mexico, Av. Universidad #3000, Delegacion Coyoacan, Col. Ciudad Universitaria, Ciudad de Mexico 04510, Mexico; tzayhriosoriogallardo@gmail.com

**Keywords:** drug repurposing, nonsteroidal anti-inflammatory drugs, NSAIDs, diabetes mellitus, dipeptidyl peptidase-4 inhibitors, DPP-4

## Abstract

A drug repurposing strategy could be a potential approach to overcoming the economic costs for diabetes mellitus (DM) treatment incurred by most countries. DM has emerged as a global epidemic, and an increase in the outbreak has led developing countries like Mexico, India, and China to recommend a prevention method as an alternative proposed by their respective healthcare sectors. Incretin-based therapy has been successful in treating diabetes mellitus, and inhibitors like sitagliptin, vildagliptin, saxagliptin, and alogliptin belong to this category. As of now, drug repurposing strategies have not been used to identify existing therapeutics that can become dipeptidyl peptidase-4 (DPP-4) inhibitors. Hence, this work presents the use of bioinformatics tools like the Activity Atlas model, flexible molecular docking simulations, and three-dimensional reference interaction site model (3D-RISM) calculations to assist in repurposing Food and Drug Administration (FDA)-approved drugs into specific nonsteroidal anti-inflammatory medications such as DPP-4 inhibitors. Initially, the Activity Atlas model was constructed based on the top scoring DPP-4 inhibitors, and then the model was used to understand features of nonsteroidal anti-inflammatory drugs (NSAIDs) as a function of electrostatic, hydrophobic, and active shape features of DPP-4 inhibition. The FlexX algorithm was used to infer protein–ligand interacting residues, and binding energy, to predict potential draggability towards the DPP-4 mechanism of action. 3D-RISM calculations on piroxicam-bound DPP-4 were used to understand the stability of water molecules at the active site. Finally, piroxicam was chosen as the repurposing drug to become a new DPP-4 inhibitor and validated experimentally using fluorescence spectroscopy assay. These findings are novel and provide new insights into the role of piroxicam as a new lead to inhibit DPP-4 and, taking into consideration the biological half-life of piroxicam, it can be proposed as a possible therapeutic strategy for treating diabetes mellitus.

## 1. Introduction

Impairment in insulin production or improper use of insulin, or both, lead to changes in blood glucose levels resulting in the most common metabolic disorder known as diabetes mellitus (DM) [1]. Type 1 diabetes is a result of the autoimmune destruction of islet cells of the pancreas [2], whereas type 2 diabetes is a result of impaired insulin signaling and environmental factors [3]. Approximately 425 million people worldwide have type 2 diabetes, and a significant portion (11.5 million) live in Mexico [4,5]. Prevalence has increased rapidly during the past years and is expected to rise 10% by 2040 [6], as well as diabetes complications such as cardiovascular risk, diabetic retinopathy, and diabetic nephropathy [7]. The increasing pattern in prevalence has indeed increased the economic costs incurred by patients and as well as by the healthcare sector in respective countries [8]. Hence, developing countries have adopted preventive strategies like an increase in physical activity and improvements in diet [9]. Recently, glucagon-like peptide-1 receptor agonists and dipeptidyl peptidase-4 (DPP-4) inhibitors have been successful in clinical use in regulating glucose metabolism in diabetes mellitus patients apart from other therapies like insulin, sulfonylureas, biguanides, meglitinides, thiazolidinediones, and alpha-glucosidase inhibitors [10].

The mechanism of DPP-4 inhibitors is to stop the degradation of incretin hormones such as glucagon-like peptide-1 (GLP-1) and glucose-dependent insulinotropic peptide (GIP) by the DPP-4 enzyme to regulated glucose metabolism [11,12]. DPP-4 also degrades regulatory factors like chemokines and growth factors revealing the role of DPP-4 inhibitors in controlling inflammatory diseases [13]. Incretin hormones regulate glucose metabolism by stimulating insulin secretion and glucagon release and by reducing beta cell apoptosis [14]. Molecular investigations into DPP-4 inhibitors have revealed that key residues at the active site responsible for inhibition are hydrophobic S1 pocket (Tyr547, Ser630, Tyr631, Val656, Trp659, Tyr662, Tyr666, Asn710, Val711, and His 740) and hydrophobic S2 pocket (Glu205, Glu206, and Tyr662; S3 consists of Ser209, Arg358, and Phe357) [15]. Side effects like pancreatitis and hepatic and renal toxicity are pervasive due to Food and Drug Administration (FDA)-approved DPP-4 inhibitors in clinical use for chemicals such as sitagliptin, vildagliptin, alogliptin, and saxagliptin [10]. During treatment, complications such as diabetic retinopathy and painful diabetic nephropathy have been observed in association with aging and have been seen more in insulin-dependent diabetes [16]. Medications like opioids, tapentadol, and lidocaine are recommended for patients with painful diabetic nephropathy [17]. Combination therapies to treat diabetes mellitus were successful in clinical use as well as in preclinical research. The timeline to develop any chemical to reach bench to bed is almost 15–20 years and is expensive for academic institutions or industries. In addition, over the last few years, the prevalence of diabetes has been exponential, leading to higher production costs. Pain treatment for diabetes caught our attention, and successful clinical use led us to hypothesize that pain-related drugs alone can have an impact on treating diabetes mellitus [18]. It is well known that nonsteroidal anti-inflammatory drugs (NSAIDs) in clinical use have been proven to act by inhibiting cyclooxygenase (COX) enzymes; however, some of these drugs have the risk of kidney failure, and NSAIDs are generally not used in the case of diabetes mellitus [19]. Salsalate and ibuprofen drugs are used to treat arthritis and have been proven to reduce blood glucose levels by decreasing inflammatory mediators in type 2 diabetes patients, which demonstrates the potential use of NSAIDs alone to lower blood glucose levels [20,21].

The literature has revealed that drug repurposing is a new strategy used by academia and pharmaceutical industries to identify new uses for approved or investigational drugs [22]. Numerous advantages associated with drug repurposing are lower risk failure considering toxicity in humans, reduction in the timeline for drug development, a small investment in comparison to new chemical studies, a rapid return on investment of repurposed drugs, and the understanding of new therapeutic targeted pathways [23]. Blind-based, target-based, knowledge-based, signature-based, pathway- or network-based and targeted mechanism-based are some of the existing methods to apply drug-repositioning technology [24]. The abovementioned drug repurposing methods have been applied to identify the drug’s new role, and Table 1 lists some of the repositioned compounds and the compound approval by the FDA. These technologies are successfully employed in cancer [25,26] and viruses [27,28] and leave scope for academic investigators to apply for more diseases. Recently, Goldstein et al. (2018) studied calcium channel blockers as repurposing drugs to benefit gestational diabetes [29]. Another study by Oral et al. (2017) repurposed an anti-asthma drug to improve glucose levels in patients with diabetes mellitus [30].

As of today, no studies have been reported on drug repurposing as a strategy to identify existing drugs as alternative therapeutics to inhibit DPP-4 and as well as regulate glucose metabolism. Therefore, the present study is focused on using drug repurposing technology, specifically the Activity Atlas model screening of FDA-approved drugs and, in particular, nonsteroidal anti-inflammatory drugs (NSAIDs) are studied. Molecular docking simulations using FlexX was used to predict protein–ligand interactions. The best compound was chosen based on features revealed by the Activity Atlas model, binding energy, availability and cost of drugs in Mexico, and less toxicity profile in humans. Lastly, the best FDA-approved NSAID discovered using computational methodology was validated experimentally in vitro to demonstrate the role of repurposing drugs in regulating glucose metabolism and thereby treating diabetes mellitus patients.

## 2. Materials and Methods

### 2.1. Chemicals and Reagents

The chemical piroxicam, dimethyl sulfoxide, and the human dipeptidyl peptidase-4 enzyme inhibitor screening kit were purchased from Sigma-Aldrich (St. Louis, MO, USA).

### 2.2. Compound Alignment, SAR Development, and Visualization of the Activity Atlas Model

The Activity Atlas model provided by Forge Software (Cresset Inc., Cambridgeshire, UK) was used to create a qualitative structure–activity Relationship (SAR) model to aid in understanding the common features, such as positive and negative electrostatics, favorable and unfavorable hydrophobic features, and shapes of activity of a total of 107 DPP-4 inhibitors downloaded from the BindingDB database [31] (https://www.bindingdb.org/bind/index.jsp). Chemicals in SMILES format are shown in Tables S1 and S2, Supplementary Materials. The resulting Activity Atlas model was employed to understand the potential of FDA-approved nonsteroidal anti-inflammatory drugs (NSAIDs) possessing features similar to DPP-4 inhibitors. The key features were visualized using the Forge visualization tool (Version 2.0, Cresset Inc., Cambridgeshire, UK).

### 2.3. DPP-4 Protein–FDA-Approved NSAID Molecular Docking Simulations

Flexible docking calculation was carried out to simulate the interactions between NSAIDs and DPP-4. For this purpose, FDA-approved NSAIDs were chosen to do docking simulations using the FlexX docking algorithm [32] provided by LeadIT software package version 2.3.2 (BioSolveIT GmbH, Sankt Augustin, Germany). The X-ray crystal structure of vildagliptin bound to the DPP-4 complex was retrieved from the Protein Data Bank (https://www.rcsb.org/) whose PDB ID is 6B1E. Initially, the complex was validated using the structure validation server SAVES (http://servicesn.mbi.ucla.edu/SAVES/), and then the complex was loaded into the protein preparation wizard provided by the FlexX software. The binding site was constructed around vildagliptin at the DPP-4 protein (Chain A was chosen), and then the docking strategy was performed on chosen NSAIDs. The FlexX scoring function was used to calculate energy, and the lowest energy resulting structure was selected as the final structure to study the key interacting amino acid residues of DPP-4 involved in interactions with NSAIDs. Discovery Studio Visualizer (Biovia, San Diego, CA, USA, 2018) was used to understand and visualize interactions among DPP-4 and NSAIDs.

### 2.4. 3D-RISM Analysis to Investigate NSAID and DPP-4 Interactions

Flare Software provides a module to use the reference interaction site model (RISM), a modern approach to solvation based on the molecular Ornstein–Zernike equation [33,34]. Three-dimensional reference interaction site model (3D-RISM) is a novel method to investigate the location and stability of water molecules in the DPP-4 protein and the druggable region. In this study, a 3D-RISM analysis was carried out on the binding poses of NSAIDs and DPP-4 to investigate the stability of water molecules surrounding the inhibitor and the active site of DPP-4.

### 2.5. In Vitro DPP-4 Inhibition Assay

In vitro DPP-4 inhibition assay was performed by using the human dipeptidyl peptidase-4 inhibitor screening kit (Sigma-Aldrich, St. Louis, MO, USA). The central principle of the assay is based on the cleavage of the fluorescent product Gly-Pro (λex = 360 nm and λem= 460 nm) by the DPP-4 protein present in the well, and fluorescence was monitored using a microwell plate reader VICTOR Nivo™ multimode reader (Perkin Elmer, Waltham, MA, USA). The fluorescence emissions were collected for 30 min, and the percent of relative inhibition was calculated using Equation (1), where *δ*f/*δ*t represents the change in fluorescence over the desired interval. The data shown in graphs were expressed as mean ± standard deviation (SD) of duplicate experiments, and unpaired *t*-test was used to calculate significant statistical difference at *p* < 0.05. GraphPad software (Version 6, GraphPad Software Inc., San Diego, CA, USA) was used to make graphs and calculations.

(1) $$Relative\;percent\;of\;DPP-4\;inhibition=\;\left(\frac{\left(\frac{\delta f}{\delta t}\right)DPP-4\;protein-\left(\frac{\delta f}{\delta t}\right)DPP-4\;protein\;inhibitor}{\left(\frac{\delta f}{\delta t}\right)DPP4\;protein}\right)\times 100$$

## 3. Results and Discussion

### 3.1. Activity Atlas Model Reveals the Structure–Activity Relationship (SAR) Mechanism

**Figure 1:**
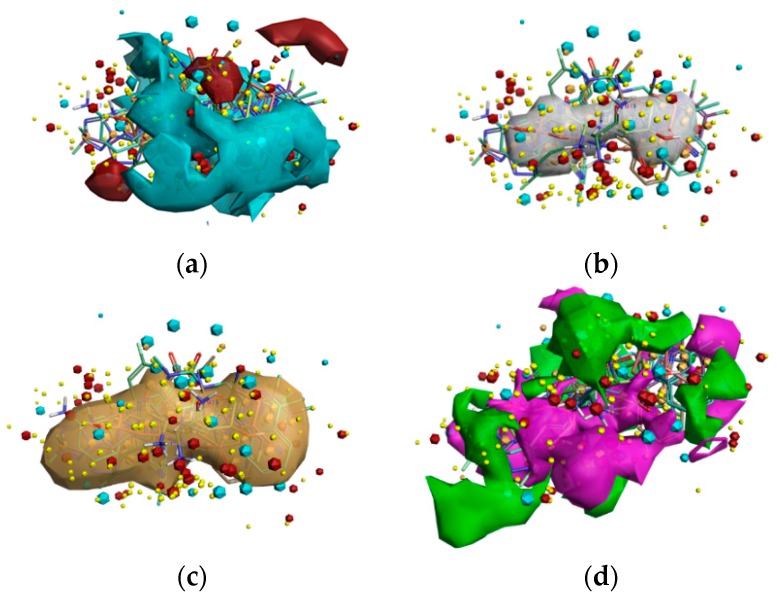
The Activity Atlas model is revealing insights into DPP-4 inhibitory activity. (**a**) Red shows the positive electrostatic field, and blue presents the negative electrostatic field responsible for the inhibition activity of the DPP-4 enzyme. (**b**) White exhibits the average shape responsible for the inhibitory activity. (**c**) Brown designates the hydrophobic shape features for inhibition. (**d**) Inhibition favorable region is shown in green, and unfavorable region shape is shown in purple.

To reveal the critical features of DPP-4 inhibition activity and apply nonsteroidal anti-inflammatory drug repurposing, SAR was performed using the Activity Atlas model; studies related to an average of actives and activity cliff summary were studied for DPP-4 inhibitors in clinical use and preclinical studies. A total of 99 DPP-4 inhibitors (supplementary data in Table S2, Supplementary Materials), together with their 50% inhibitory concentration values, were retrieved from the BindingDB database and aligned with the reference template created based on eight DPP-4 inhibitors in clinical use. The generated Activity Atlas model showed the characteristics of the DPP-4 inhibitors as a function of the average of actives and activity cliff summary for the DPP-4 inhibitors in clinical use and preclinical studies. Figure 1 shows the average of actives, regions of activity, and activity cliff summary of a total of 107 DPP-4 inhibitors. Red sites in Figure 1a are responsible for DPP-4 inhibition, and positive field regions are visualized within the active SAR model. The shape of activities responsible for DPP-4 inhibition is represented in white in Figure 1b, and hydrophobic regions accountable for DPP-4 inhibition, are reported in brown (Figure 1c). Moreover, the model revealed favorable (green) and unfavorable (purple) shape features responsible for DPP-4 inhibition (shown in Figure 1d). Finally, in brief, more significant red and cyan regions indicate higher DPP-4 inhibitory activity.

In order to identify the potential of 20 NSAIDs possessing molecular characteristics similar to existing DPP-4 inhibitors, field-based screening was performed using field template alignment, and novelty scores were calculated. Initially, 20 NSAID chemical structures were downloaded from the PubChem tool. All 20 chemicals were aligned to the created Activity Atlas model to establish a score, with a novelty score of “low, high, and very high”. The novelty score assigned by the Activity Atlas model was the result of assessing how the different chemicals were chosen and of the training data used in constructing the activity model (Table 2). All 20 molecules were considered for the molecular docking studies to understand protein–ligand interactions.

In general, the Activity Atlas method used in this study can be applied to more massive datasets to obtain a single picture, that summarizes SAR into 3D maps of molecular features, and to help with the design of new compounds as drugs and the optimization of existing drugs. The Activity Atlas model provides a global qualitative view about molecular features as a function of electrostatic, hydrophobic, and shape properties qualitatively. The methodology used in this study can be automated and run on servers to manipulate large datasets (more than 1000). The program supports Python and Workflows that can be integrated, so the proposed method applies to any drug target of interest.

### 3.2. Molecular Docking Simulations

**Figure 2:**
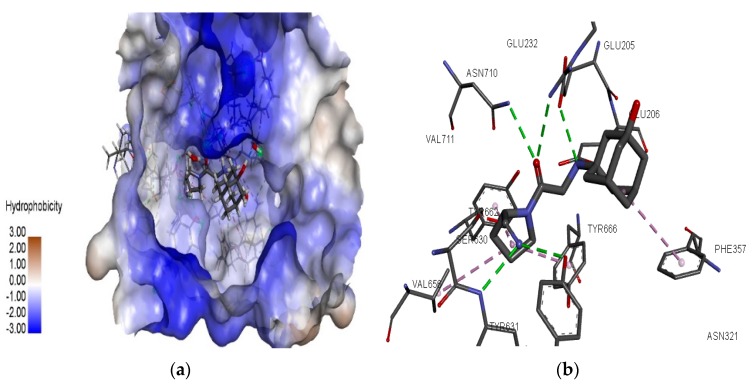
The binding site of vildagliptin at DPP-4 active site. (**a**) The binding site constructed around the active site of DPP-4 using LeadIT software. (**b**) Two-dimensional DPP-4 protein–vildagliptin binding pocket interaction analysis visualized using Discovery Studio Visualizer.

In this study, the molecular docking tool FlexX provided by LeadIT software was employed. The redocking methodology was used to validate the docking protocol used in this study. It was observed that the FlexX docking algorithm could replicate experimental binding poses and the interacting residues similar to X-ray crystal structures of vildagliptin (6B1E), saxagliptin (3BJM), and sitagliptin (4FFW). The binding site was chosen around vildagliptin (shown in Figure 2a) and interacting residues involved at the binding site of DPP-4 (shown in Figure 2b). The resulting structure was used to perform FlexX docking of 20 NSAID compounds. The FlexX scoring function calculated energy values of the 10 different poses of each of the 20 compounds and the docking strategy was chosen as ligand-driven by enthalpy and entropy (hybrid approach). The lowest energy was selected as the best binding pose, and the interacting residues in the binding site of the DPP-4 proteins were manually compared to the results of the activity cliff summary of the compounds, chosen to select the best NSAID compound with the potential for DPP-4 inhibition.

Twenty NSAIDs were studied for protein–ligand interactions using the FlexX algorithm, and the binding free energy analysis revealed the top five compounds possessing an excellent binding affinity to DPP-4. The top compounds were mefenamic acid, ketoprofen, piroxicam, meloxicam, and tolmetin. Basic interacting residues involved at the binding site were observed, and further interaction analysis by Discovery Studio Visualizer allowed us to study pi–cation, pi–alkyl, and hydrogen bond interactions, van der Waals interactions, as well as pi–pi stacked, pi–T-shaped, and pi–anion interactions with amino acids at the druggable region of DPP-4.

The successful hit NSAID drug mefenamic acid possesses the highest FlexX score of −26.6 kJ/mol and the interacting residues involved in the binding site are TYR 547, ARG 125, GLU 205, ASN 710, TYR 662, VAL 656, SER 630, and ASN 710. Further non-bonding interaction analysis of the mefenamic acid and DPP-4 active site revealed the following: strong hydrogen bonding with residues TYR 547, SER 630, and ASN 710; strong pi–cation interactions by ARG 125 and GLU 205; van der Waals interactions by TYR 662 and VAL 656; and pi–alkyl interactions by TYR666 at the druggable region of DPP-4 (shown in Figure S1a, Supplementary Materials). The biological half-life time of mefenamic acid is approximately 2 h [35].

Similarly, another top scoring successful NSAID, ketoprofen, possesses a FlexX score of −20.9 kJ/mol and the interacting amino acids involved in the binding site are TYR 662, ASN 710, TYR 66, TRP 659, SER 630, VAL 711, HIS 740, TYR 547, ARG 125, GLU 205, and ASN 710. Non-bonding interaction analysis of the ketoprofen and DPP-4 active site revealed the following: strong hydrogen bonding by amino acids TYR 662 and ASN 710; pi–pi stacked and T-shaped interactions by TYR 666 and TYR 547; and pi–anion interactions by GLU 205 and ARG 125 at the druggable region of DPP-4 (shown in Figure S1b, Supplementary Materials). The biological half-life time of ketoprofen is 1–3 h [36].

Meloxicam, another NSAID, binds similarly to mefenamic acid and ketoprofen and possesses a FlexX score of −18.8 kJ/mol. PHE 357, GLU 206, TYR 547, ARG 125, ASN 710, GLU 205, TYR 662, SER 630, VAL 711, VAL 656, and TYR 666 are the interacting residues involved in the binding site of DPP-4 in complex with meloxicam. Non-bonding interaction analysis of the meloxicam and DPP-4 active site revealed the following: hydrogen bond interactions by amino acids TYR 547, ARG 125, and ASN 710; pi–sulfur and pi–pi interactions with PHE 357; and water hydrogen bonding at the druggable site of DPP-4 (shown in Figure S1c, Supplementary Materials). The biological half-life time of meloxicam is 20 h [37].

**Figure 3:**
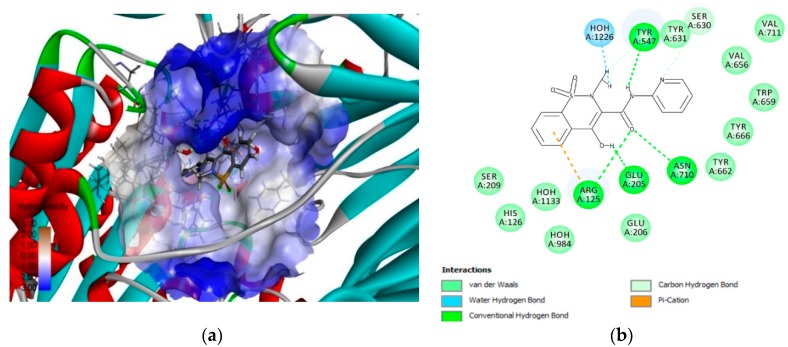
DPP-4 interactions with piroxicam generated using the FlexX docking algorithm. (**a**) The binding pose view of piroxicam at the active site of DPP-4 and the hydrophobic surface visualized around the active site. (**b**) Binding mode of piroxicam interaction in the active site of DPP-4 (6b1E). The interacting residues are depicted with different colors: van der Waals (light green dotted line), pi–cation (orange), hydrogen bonding (dark green) and, finally, water hydrogen bond (light blue).

Tolmetin possesses a FlexX score of −18.0 kJ/mol and interacting residues involved at the binding site are TYR 547, ASN 710, GLU 205, ARG 125, TYR 631, SER 630, TYR 662, and VAL 656. In addition, non-bonding interaction analysis of the tolmetin and DPP-4 active site revealed the following: hydrogen bond interactions by amino acids TYR 547, ARG 125, and GLU 205; pi–cation interactions with ARG 125; carbon–hydrogen bond with SER 630; and water hydrogen bonding at the druggable site of DPP-4 (shown in Figure S1d, Supplementary Materials). The biological half-life time of tolmetin is 0.83 h [38]. Certain additional filters such as the NSAIDs’ availability for clinical use, toxicity-related studies based on the literature, cost, and the biological half-life time were used to choose a potential NSAID drug to be evaluated experimentally and to demonstrate the application of the SAR model and the FlexX molecular docking studies. Finally, based on the filters, piroxicam was selected from the remaining 19 NSAIDs and the top five scoring NSAIDs because molecular docking simulation studies revealed that piroxicam possesses a FlexX score of −19.9 kJ/mol. The binding pose was visualized using the Discovery Studio Visualizer, which is shown in Figure 3. The compound does not possess any unfavorable interactions with water molecules and amino acids at the active site as was seen in the case of the NSAIDs mefenamic acid and ketoprofen (shown in Figure S1, Supplementary Materials). The binding free energy scores of the 20 NSAIDs and novelty scores calculated using the Activity Atlas model are shown in Table 2.

The interacting residues involved in the binding site of the DPP-4 protein with piroxicam were TYR 547, ARG 125, GLU 206, ASN 710, TYR 662, VAL 656, and SER 630 (shown in Figure 3b). It is well known that critical regions responsible for DPP-4 inhibition contain hydrophobic S1 pocket (TYR 662), N-terminal recognition region of DPP-4 (GLU 205 and GLU 206), and hydrophobic S2 pocket (TYR 547 and ASN 710) and that the enzyme catalytic residue (SER630) belongs to the druggable region. Interaction analysis of the piroxicam and DPP-4 active site revealed the following: strong hydrogen bonding by amino acids ARG 125, GLU 205, ASN 710, and TYR 547; strong pi–cation interactions by ARG 125; and van der Waals interactions by TYR 662, Glu 206, and SER 630 at the druggable region of DPP-4.

One point that is important to consider is that piroxicam (20 mg dosage) is available in Mexico for clinical use at the cost of 3.0 USD (data retrieved from San Pablo Farmacia, https://www.farmaciasanpablo.com.mx/), compared with the prices of existing DPP-4 inhibitors like sitagliptin (25 mg dosage, 56.59 USD), vildagliptin (50 mg dosage, 22.0 USD), saxagliptin (5 mg dosage, 59.70 USD), linagliptin (5 mg dosage, 20.92 USD), and alogliptin (25 mg dosage, 38.11), which are significantly higher in price. Table S3 provides the information on FDA-approved NSAIDs and their retail prices in Mexico.

**Figure 4:**
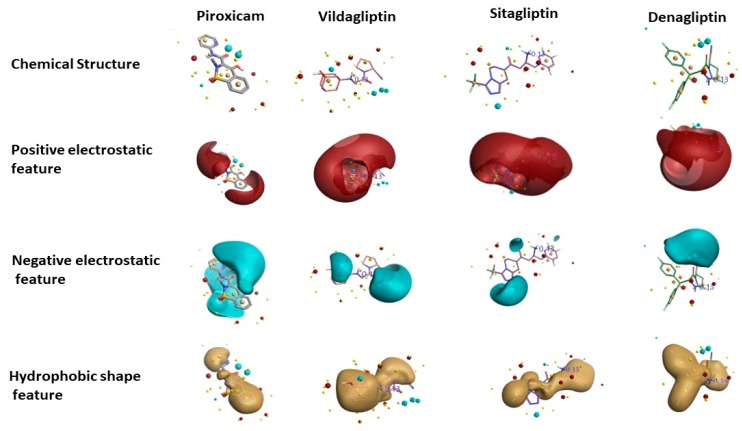
Prediction of piroxicam molecular features: positive electrostatics, negative electrostatics, and hydrophobic shape calculated using XED force field, provided by the Activity Atlas model in comparison with DPP-4 inhibitors in clinical use.

Figure 4 shows the results of the Activity Atlas model describing the 3D representations of the four different positive (red) and negative (blue) electrostatic and hydrophobic shape (brown or gold) features calculated using the Cresset XED force field of piroxicam in comparison with existing FDA-approved DPP-4 inhibitors: vildagliptin, sitagliptin, and denagliptin. Figure 4 shows that some features of positive electrostatic shape are conserved in the case of piroxicam and DPP-4 inhibitors. Regarding the negative electrostatic feature, piroxicam shares some features similar to sitagliptin and denagliptin. The hydrophobic shape of piroxicam in the central region is common to all three DPP-4 inhibitors, and it shares the same feature in the upper left region with denagliptin. In addition, when field differences were examined, it resulted in a distinct negative electrostatic and hydrophobic feature of piroxicam compared with the other DPP-4 inhibitors, which might influence the inhibition activity either positively or negatively, and therefore it had to be evaluated experimentally.

### 3.3. 3D-RISM Analysis of DPP-4–Piroxicam

**Figure 5:**
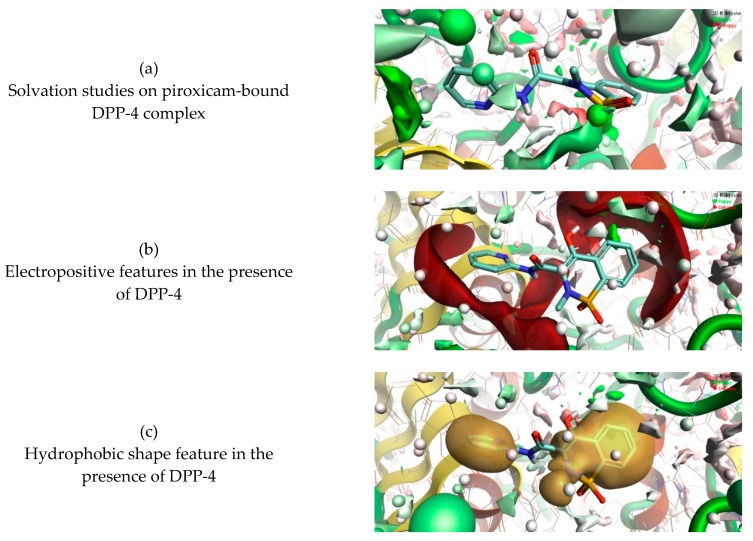
Three-dimensional reference interaction site model (3D-RISM) analysis to understand the stability of water molecules at the piroxicam-bound DPP-4 proteins (**a**) The 3D-RISM run analysis reveals oxygen isodensity surface at *ρ* = 5 and localized 3D-RISM waters, colored by ΔG. (**b**) Electropositive surface features (red) of the NSAID drug piroxicam in the presence of DPP-4 and bulky water. (**c**) Hydrophobic shape features (brown or gold) of the NSAID drug piroxicam in the presence of DPP-4 and bulky water.

The complex formed by piroxicam and the DPP-4 protein as predicted by the FlexX algorithm was used as starting macromolecule to study the stability of water molecules in the active site using 3D-RISM. At the end of a 3D-RISM run, the water molecules in the DPP-4 formed a high-water density. The water density was colored according to the calculated ΔG for each water molecule, averaged over all orientations. Figure 5 shows the oxygen density surface of piroxicam bound to the enzyme DPP-4 with favorable (green) and unfavorable water (red). This reveals the stability of each water molecule relative to bulk water at the active site of DPP-4. Green reveals the more stable behavior of the water molecules and which are more difficult to be displaced by piroxicam. Similarly, red reveals the less stable behavior of the water molecules which are more easily displaced by piroxicam (Figure 5a). The study of the favorable and unfavorable water molecules improved the precision of how piroxicam binds at the active site of DPP-4. Figure 5b,c shows the electropositive and hydrophobic shape molecular features of piroxicam in the presence of the DPP-4 protein and high-water density. The information gained from the 3D-RISM calculations can be used to perform piroxicam modifications, in order to improve the potency of DPP-4 inhibition.

### 3.4. In Vitro Assay of Piroxicam Inhibition of Human Dipeptidyl Peptidase-4

**Figure 6:**
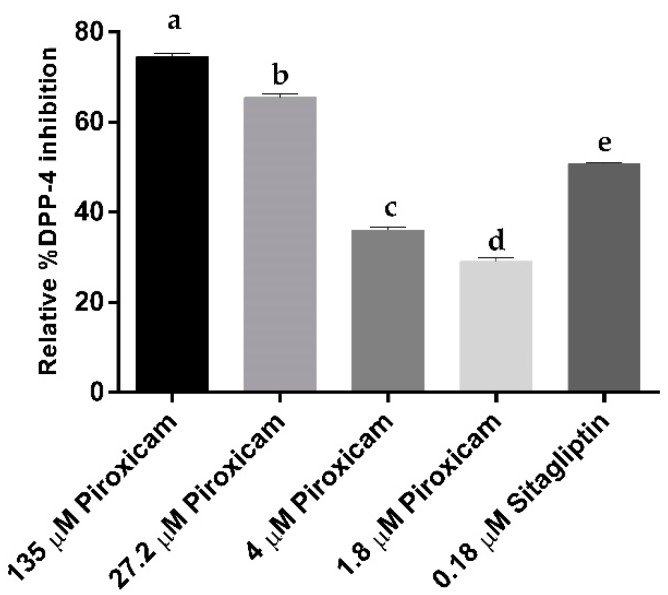
Comparison of relative percentages of DPP-4 inhibition by piroxicam at concentrations of 135 μM, 27.2 μM, 4 μM, and 1.8 μM, and by sitagliptin at a concentration of 0.018 µM, respectively. Means with different letters are significantly different at *p* < 0.05 determined by two-tailed unpaired *t*-test.

Piroxicam was evaluated experimentally at 135 µM, 27.2 µM, 4 μM, and 1.8 μM concentrations to demonstrate the applicability of the drug repurposing NSAID as an inhibitor of DPP-4 activity. Figure 6 reveals the in vitro assay of piroxicam’s role as DPP-4 inhibitor in a concentration-dependent manner. The relative percentage of DPP-4 inhibition by piroxicam was 74.50%, 65.50%, 36.5%, and 29.60% respectively. The inhibition role was statistically significant (*p* < 0.05). Positive control sitagliptin inhibited DPP-4 activity by 51%, at a concentration of 0.018 µM (Figure 6), whereas the percentage of inhibition curve calculations revealed that piroxicam had an IC50 value of 9.9 µM. These findings are novel and the first study to demonstrate that piroxicam inhibits DPP-4. Based on these results, it can generally be concluded that NSAIDs can inhibit DPP-4. However, it must be taken into consideration that DPP-4 inhibition by piroxicam is in micromolar concentration and is less in comparison to sitagliptin and other known DPP-4 inhibitors in clinical use. Hence, we conclude that piroxicam is a promising lead compound for the development of DPP-4 inhibitors.

Nevertheless, the biological half-life time of piroxicam was about 45 h [39], whereas that of sitagliptin was about 8–14 h [40]. If one considers biological half-life time as another important parameter for drug design and discovery that directly correlates with the number of dosages [41], piroxicam alone or a derivative of piroxicam can be considered as a probabilistic future therapeutic option to treat diabetes mellitus. In addition, the inhibition of DPP-4 by piroxicam in this study explains the possible mechanism of action behind the reduction in blood sugar levels in rats and humans when treated with piroxicam [42] and could be the mechanism behind reducing diabetic neuropathy [43].

## 4. Conclusions

In this study, piroxicam was discovered as a repurposing drug to inhibit human DPP-4 protein, by combining computational approaches, the Activity Atlas model, and molecular docking simulations. The Activity Atlas model revealed similarities in positive and negative electrostatic and hydrophobic shape features between piroxicam and approved DPP-4 inhibitors. FlexX molecular docking simulations and 3D-RISM calculations revealed the binding of piroxicam in the DPP-4 druggable region. In vitro experiments on human DPP-4 inhibition assay revealed that piroxicam inhibits DPP-4 in a concentration-dependent manner with an estimated IC50 value of 9.9 µM in comparison with sitagliptin as positive control whose IC50 value is 0.018 µM. These results are novel and provide new insights into the role of piroxicam as a therapeutic strategy for treating diabetes mellitus. Future studies will focus on understanding the mechanism of piroxicam towards DPP-4 inhibition in murine models.

## Figures and Tables

**Table 1:** Drug repurposing technology applications in clinical use.

No.	Drug	New Indication	Year Approved (for New Indication)
1	Methotrexate	Rheumatoid arthritis	1999
2	Topiramate	Migraine	2004
3	Cymbalta	Diabetic peripheral neuropathy	2004
4	Mifepristone	Cushing’s syndrome	2012

**Table 2:** Food and Drug Administration (FDA)-approved nonsteroidal anti-inflammatory drugs (NSAIDs) along with FlexX scores provided by the FlexX algorithm and novelty scores calculated using the Activity Atlas model.

No.	Chemical Name	FlexX Score (kJ/mol)	Novelty Score
1	Celecoxib	−8.7	Moderate
2	Valdecoxib	−17.2	Very High
3	Rofecoxib	−10.6	High
4	Diclofenac	−16.06	High
5	Diflunisal	−15.7	High
6	Etodolac	−10.19	High
7	Fenoprofen	−13.4	Moderate
8	Flurbiprofen	−15.4	High
9	Ibuprofen	−16.06	Moderate
10	Indomethacin	−13.47	Very High
11	Ketoprofen	−20.9	Very High
12	Ketorolac	−17.7	Very High
13	Mefenamic Acid	−26.680	Low
14	Meloxicam	−18.9	Low
15	Nabumetone	−13.0	Low
16	Naproxen	−15.4	Moderate
17	Oxaprozin	−8.5	Very High
18	Piroxicam	−19.9	Low
19	Sulindac	−8.4	Very High
20	Tolmetin	−18.0	Very High

## Supplementary Materials

The following are available online at https://www.mdpi.com/1999-4923/11/5/238/s1, Table S1: FDA-approved DPP-4 inhibitors and reference dataset used in constructing the Activity Atlas model, Table S2: DPP-4 inhibitors used to make the Activity Atlas model, Table S3: FDA-approved NSAIDs retail prices in Mexico, Figure S1: Two-dimensional ligand interactions with DPP-4 protein (PDB ID:6B1E). Non-bonded are depicted with different colors, and Discovery Studio Visualizer was used for the visualization of interactions.
