# Supplementary material for: Repurposing of FDA-Approved NSAIDs for DPP-4 Inhibition as an Alternative for Diabetes Mellitus Treatment: Computational and in Vitro Study
Source: Pharmaceutics. 2019 May 17;11(5):238. doi: 10.3390/pharmaceutics11050238 (PMC6572294; doi:10.3390/pharmaceutics11050238)
Supplement: Supplementary file 1 [file pharmaceutics-11-00238-s001.pdf]

pharmaceutics

Supplementary Materials: Repurposing of FDA-Approved NSAIDs for DPP4 Inhibition as an Alternative for Diabetes Mellitus Treatment: Computational and in Vitro Study

Veera C.S.R Chittepu, Poonam Kalhotra, Tzayhri Osorio Gallardo, Tzayhri Gallardo-Velázquez and Guillermo Osorio-Revilla

Table S1. FDA-approved DPP-4 inhibitors and reference dataset used in constructing the Activity Atlas model.

Name	SMILE	IC50 nM
Alogliptin	CN1C(=O)C=C(N(C1=O)CC2=CC=CC=C2C#N)N3CCCC(C3)N	7
Denagliptin	C1C(CN(C1C#N)C(=O)C(C(C2=CC=C(C=C2)F)C3=CC=C(C=C3)F)N)F	22
Linagliptin	CC#CCN1C2=C(N=C1N3CCCC(C3)N)N(C(=O)N(C2=O)CC4=NC5=CC=CC=C5C(=N4)C)C	1
Melogliptin	C1CC(CC1CN2C=NC=N2)NCC(=O)N3CC(CC3C#N)F	1.61
Saxagliptin	C1C2CC2N(C1C#N)C(=O)C(C34CC5CC(C3)CC(C5)(C4)O)N	3.37
Sitagliptin	C1CN2C(=NN=C2C(F)(F)F)CN1C(=O)CC(CC3=CC(=C(C=C3F)F)F)N	18
Vildagliptin	C1CC(N(C1)C(=O)CNC23CC4CC(C2)CC(C4)(C3)O)C#N	3.5

Table S2. DPP-4 inhibitors used to make the Activity Atlas model.

No.	PubChemm CID	SMILE	IC50 μM
1	10250452	CN(C)C(=O)[C@H]([C@H](N)C(=O)N1CC[C@H](F)C1)c1ccc(cc1)-c1ccc(F)cc1	0.012
2	72703825	CC#CCn1c(nc2n(C)c(=O)n(Cc3nc4cc(F)ccc4s3)c(=O)c12)N1CCC[C@@H](N)C1	0.05
3	9884543	C[C@H]([C@H](N)C(=O)N1CC[C@H](F)C1)c1ccc(cc1)-c1ccc(F)cc1	0.064
4	72703825	CC#CCn1c(nc2n(C)c(=O)n(Cc3nc4cc(F)ccc4s3)c(=O)c12)N1CCC[C@@H](N)C1	0.08
5	72703824	CC#CCn1c(nc2n(C)c(=O)n(Cc3nc4ccccc4s3)c(=O)c12)N1CCC[C@@H](N)C1	0.1
6	6918572	CC(C)[C@H](N)C(=O)N1CCC[C@H]1B(O)O	0.1
7	44394157	COc1cc(OC)cc(c1)-c1nc(N(C)C)c(CN(C)C)c(n1)-c1ccc(Cl)cc1Cl	0.1
8	448436	COc1cc(OC)cc(c1)-c1nc(N)c(CN)c(n1)-c1ccc(Cl)cc1Cl	0.1
9	10096344 (Linagliptin)	CC#CCn1c(nc2n(C)c(=O)n(Cc3nc(C)c4ccccc4n3)c(=O)c12)N1CCC[C@@H](N)C1	0.1
10	24798754	N[C@H]1C[C@H](CO[C@@H]1c1cc(F)ccc1F)N1Cc2nn3nccnc3c2C1	0.12
11	11163434	O=C([C@@H]1C[C@@H](CN1)Nc1ccc(C#N)c(c1)C#N)N1CCC[C@H]1C#N	0.13
12	9821205	Clc1cc(N[C@@H]2CN[C@@H](C2)C(=O)N2CCC[C@H]2C#N)ccc1C#N	0.13
13	68658979	Cc1cc2ncc3CN(Cc3n2n1)[C@H]1CC[C@@H]([C@@H](N)C1)c1cc(F)c(F)cc1F	0.14
14	68269333	N[C@H]1C[C@H](CSC1c1cc(F)ccc1F)N1Cc2n[nH]c(C(N)=O)c2C1	0.17
15	24776939	N[C@@H](CC(=O)N1CCn2c(nnc2C(F)(F)F)[C@H]1Cc1ccc(F)cc1)Cc1cc(F)c(F)cc1F	0.18
16	10109454	[O-][N+](=O)c1ccc(N[C@@H]2CN[C@@H](C2)C(=O)N2CCC[C@H]2C#N)cc1	0.18
17	44400485	O=C([C@@H]1C[C@@H](CN1)Nc1ccc(cc1)C#N)N1CCC[C@H]1C#N	0.19
18	72551282	CC#CCn1c(nc2n(C)c(=O)n(Cc3nc4cc(Cl)ccc4s3)c(=O)c12)N1CCC[C@@H](N)C1	0.2
19	67507176	O=C([C@@H]1C[C@@H](CN1)N1CCN(CC1)c1nnnn1-c1ccccc1)N1CCSC1	0.2
20	10286977	NCc1c(N)nc(nc1-c1ccc(Cl)cc1Cl)-c1cccc(F)c1	0.2
21	10126186	NCc1c(N)nc(nc1-c1ccc(Cl)cc1Cl)-c1ccc(F)cc1	0.2
22	44453922	CS(=O)(=O)c1ccc(-c2noc(n2)[C@@H](CC2CC2)[C@H](N)C(F)=C2CCCC2)c(Cl)c1	0.21
23	127052637	Cc1nc2ncc3CN(Cc3n2n1)[C@H]1CC[C@@H]([C@@H](N)C1)c1cc(F)c(F)cc1F	0.23
24	127052638	N[C@H]1C[C@H](CC[C@@H]1c1cc(F)c(F)cc1F)N1Cc2cnc3nnc(C4CC4)n3c2C1	0.24
25	44400445	O=C([C@@H]1C[C@@H](CN1)Nc1ccc(cn1)C#N)N1CCC[C@H]1C#N	0.25
26	67507913	O=C([C@@H]1C[C@@H](CN1)N1CCN(CC1)c1nccn1-c1ccccc1)N1CCSC1	0.26
27	10149048	C[C@H](N)C(=O)N1CCC[C@H]1B(O)O	0.26
28	44400473	COc1ccc(N[C@@H]2CN[C@@H](C2)C(=O)N2CCC[C@H]2C#N)cc1OC	0.28
29	23646452	N[C@@H](CC(=O)N1CCCNC(=O)[C@H]1Cn1cccn1)Cc1cc(F)c(F)cc1F	0.29
30	70690676	N[C@@H](CCCN=C(N)N)C(=O)N1C[C@@H](O)C[C@H]1B(O)O	0.3
31	68416066	Cc1cc(N2CCN(CC2)[C@@H]2CN[C@@H](C2)C(=O)N2CCSC2)n(n1)-c1cccc(F)c1	0.3
32	44394364	CC(C)[C@H](Oc1ccc(CNC(=O)[C@@H]2CSCN2C(=O)CC([NH3+])Cc2cc(F)ccc2F)cc1) C(O)=O	0.3
33	9942554	CC(C)[C@@H](N)C(=O)N1CCC[C@H]1B(O)O	0.3

34	10798659	CC(C)[C@H](N)C(=O)N1CCC[C@@H]1B(O)O	0.3
35	44445057	N[C@@H](CC(=O)N1CCn2c(nnc2C(F)(F)F)C1Cc1ccccc1C(F)(F)F)Cc1cc(F)c(F)cc1F	0.31
36	44445063	N[C@@H](CC(=O)N1CCn2c(nnc2C(F)(F)F)C1C(O)c1ccc(F)cc1)Cc1cc(F)c(F)cc1F	0.32
37	71460143	FC(F)(F)c1cc(N2CCN(CC2)[C@@H]2CN[C@@H](C2)C(=O)N2CCSC2)n(n1)-c1ccccc1	0.32
38	44400447	COc1ccc(N[C@@H]2CN[C@@H](C2)C(=O)N2CCC[C@H]2C#N)cc1	0.33
39	49785099	N[C@@H]1CCCN(C1)c1nc2ccsc2c(=O)n1Cc1ccccc1C#N	0.33
40	56661748	Cn1c2cc(N3CCC[C@@H](N)C3)n(Cc3cc(F)ccc3C#N)c2c(=O)n(C)c1=O	0.34
41	57689709	C[C@H](N)C(=S)N1CCC[C@H]1B(O)O	0.35
42	57525787	FC(F)(F)c1cc(N2CCN(CC2)[C@@H]2CN[C@@H](C2)C(=O)N2CCSC2)c2ccccc2n1	0.37
43	11949652 (Tenegliptin)	Cc1cc(N2CCN(CC2)[C@@H]2CN[C@@H](C2)C(=O)N2CCSC2)n(n1)-c1ccccc1	0.37
44	42608447	CS(=O)(=O)c1cccc(c1)C(=O)NC[C@@H]1CCCN1C(=O)C[C@H](N)Cc1cccc(Cl)c1	0.38
45	57378228	CC#CCn1c(nc2N3CCN=C3N(Cc3nc(C)c4ccccc4n3)C(=O)c12)N1CCC[C@@H](N)C1	0.38
46	66559300	O=C([C@@H]1C[C@@H](CN1)N1CCN(CC1)c1nc2ccc(cc2[nH]1)C#N)N1CCSC1	0.39
47	44445067	N[C@@H](CC(=O)N1CCn2c(nnc2C(F)(F)F)C1Cc1ccccn1)Cc1cc(F)c(F)cc1F	0.4
48	67977924	C[C@H](F)CN1C[C@@H](C[C@H](N)C1c1cc(F)ccc1F)N1Cc2cn(nc2C1)S(C)(=O)=O	0.4
49	66559378	O=C([C@@H]1C[C@@H](CN1)N1CCN(CC1)c1nc2cc(ccc2s1)C#N)N1CCSC1	0.42
50	44445055	COc1ccc(CC2N(CCn3c2nnc3C(F)(F)F)C(=O)C[C@H](N)Cc2cc(F)c(F)cc2F)cc1	0.43
51	68269332	N[C@H]1C[C@H](CSC1c1cc(F)ccc1F)N1Cc2n[nH]c(C#N)c2C1	0.46
52	44445060	N[C@@H](CC(=O)N1CCn2c(nnc2C(F)(F)F)C1Cc1ccccc1F)Cc1cc(F)c(F)cc1F	0.46
53	10439426	CC(C)[C@@H](Oc1ccc(CNC(=O)[C@@H]2CCCN2C(=O)CC([NH3+])Cc2cc(F)ccc2F)cc 1)C(O)=O	0.48
54	58874273	Cn1c2cc(ccc2c2nc(N3CCC[C@@H](N)C3)n(Cc3cc(F)ccc3Cl)c2c1=O)C(O)=O	0.48
55	23646451	N[C@@H](CC(=O)N1CCCNC(=O)[C@H]1Cc1ccccn1)Cc1cc(F)c(F)cc1F	0.49
56	70937923	CS(=O)(=O)n1cc2CN(Cc2n1)[C@H]1CSC([C@@H](N)C1)c1cc(F)cc(F)c1F	0.5
57	54670915	Cc1c2CN(Cc2nn1S(=O)(=O)C1(C)CC1)[C@H]1CCO[C@@H]([C@@H](N)C1)c1cc(F)cc c1F	0.5
58	66559376	O=C([C@@H]1C[C@@H](CN1)N1CCN(CC1)c1nc2cc(ccc2o1)C#N)N1CCSC1	0.5
59	127050132	Cc1ccn2nc3CN(Cc3c2n1)[C@H]1CO[C@@H]([C@@H](N)C1)c1cc(F)c(F)cc1F	0.5
60	23633277	CC(C)=CCn1c(N2CCC[C@H](N)C2)c(C#N)c2ncn(Cc3nccc4ccccc34)c(=O)c12	0.5
61	57395248	COc1cccc(c1)C(=O)Cn1c(=O)n(C)c2c(C#N)c(N3CCC[C@H](N)C3)n(CC=C(C)C)c2c1= O	0.5
62	46229811	COc1cc2CCN3C[C@@H](C(N)C[C@H]3c2cc1OC)c1cccc(CF)c1	0.5
63	127049507	Cc1cc(O)n2nc3CN(Cc3c2n1)[C@H]1CO[C@@H]([C@@H](N)C1)c1cc(F)c(F)cc1F	0.5
64	66560609	O=C([C@@H]1C[C@@H](CN1)N1CCN(CC1)c1ncc(C#N)c2ccccc12)N1CCSC1	0.51
65	44400625	O=C([C@@H]1C[C@@H](CN1)Nc1ccccc1)N1CCC[C@H]1C#N	0.53
66	66559377	O=C([C@@H]1C[C@@H](CN1)N1CCN(CC1)c1nc2ccccc2s1)N1CCSC1	0.55
67	58874386	Cc1ccc(F)cc1Cn1c(nc2c1c(=O)n(C)c1cc(ccc21)C(O)=O)N1CCC[C@@H](N)C1	0.55
68	66560612	Cc1cc(N2CCN(CC2)[C@@H]2CN[C@@H](C2)C(=O)N2CCSC2)c2ccccc2n1	0.56
69	127052636	Cc1nnc2ncc3CN(Cc3n12)[C@H]1CC[C@@H]([C@@H](N)C1)c1cc(F)c(F)cc1F	0.56
71	6918537 (Vildagliptin)	OC12CC3CC(C1)CC(C3)(C2)NCC(=O)N1CCC[C@H]1C#N	0.0035
72	66559299	Clc1ccc2nc([nH]c2c1)N1CCN(CC1)[C@@H]1CN[C@@H](C1)C(=O)N1CCSC1	0.59
73	127052288	Cc1cc2ncc3CN(Cc3n2n1)[C@H]1CC[C@@H]([C@@H](N)C1)c1cc(F)ccc1F	0.6
74	12069363	CC[C@@H](C)[C@H](N)C(=O)N1C[C@@H](F)C[C@H]1C#N	0.6
75	127050761	Cc1ccnc2c3CN(Cc3nn12)[C@H]1CO[C@@H]([C@@H](N)C1)c1cc(F)c(F)cc1F	0.6
76	68269331	CS(=O)(=O)n1cc2CN(Cc2n1)[C@H]1CSC([C@@H](N)C1)c1cc(F)ccc1F	0.6
77	9859384	CC[C@H](C)[C@H](N)C(=O)N1C[C@@H](F)C[C@H]1C#N	0.6
78	11243969 (Saxagliptin)	N[C@H](C(=O)N1[C@H]2C[C@H]2C[C@H]1C#N)C12CC3CC(CC(O)(C3)C1)C2	0.0337
79	137225025	Cc1cc(O)nc2c3CN(Cc3nn12)[C@H]1CO[C@@H]([C@@H](N)C1)c1cc(F)c(F)cc1F	0.6
80	66559296	Clc1ccc2c(ccnc2c1)N1CCN(CC1)[C@@H]1CN[C@@H](C1)C(=O)N1CCSC1	0.61
81	66560524	O=C([C@@H]1C[C@@H](CN1)N1CCN(CC1)c1nccc2ccccc12)N1CCSC1	0.61
82	68419860	Cc1cc(N2CCN(CC2)[C@@H]2CN[C@@H](C2)C(=O)N2CCSC2)n(n1)-c1cccnc1	0.62
83	68417211	Cc1cc(N2CCN(CC2)[C@@H]2CN[C@@H](C2)C(=O)N2CCSC2)n(n1)-c1ccccn1	0.63
84	44590183	COCCOc1ccc2nc(sc2c1)C1(CCS(=O)(=O)CC1)NC(=O)C[C@H](N)Cc1cc(Cl)ccc1F	0.64
85	44445053	N[C@@H](CC(=O)N1CCn2c(nnc2C(F)(F)F)C1Cc1ccccc1)Cc1cc(F)c(F)cc1F	0.66
86	15949418	N[C@H]1C[C@H](CC[C@@H]1c1cc(F)c(F)cc1F)N1Cc2cncnc2C1	0.67
87	11559922	CCOC(=O)c1cn2c(c(CN)c(C)nc2n1)-c1ccc(Cl)cc1Cl	0.7
88	67977920	N[C@H]1C[C@H](CNC1c1cc(F)c(F)cc1F)N1Cc2cn(nc2C1)S(=O)(=O)C1CC1	0.7
89	24951732	N[C@H]1C[C@H](CO[C@@H]1c1cc(F)ccc1F)N1Cc2nn3cccnc3c2C1	0.7
90	4369359 (Sitagliptin)	C1CN2C(=NN=C2C(F)(F)F)CN1C(=O)CC(CC3=CC(=C(C=C3F)F)F)N	0.018

Table S3. FDA-approved NSAIDs retail prices in Mexico.

No.	Chemical Name	Available in Clinical Use in Mexico (cost in USD)
1	Celecoxib	17.08
2	Valdecoxib	Not Available
3	Rofecoxib	Not Available
4	Diclofenac	2.13
5	Diflunisal	Not Available
6	Etodolac	Not Available
7	Fenoprofen	Not Available
8	Flurbiprofen	4.08
9	Ibuprofen	2.02
10	Indomethacin	8.25
11	Ketoprofen	4.78
12	Ketorolac	1.71
13	Mefenamic Acid	Not Available
14	Meloxicam	3.43
15	Nabumetone	Not available
16	Naproxen	1.56
17	Oxaprozin	Not available
18	Piroxicam	3.06
19	Sulindac	Not available
20	Tolmetin	Not available

(a) Mefenamic acid

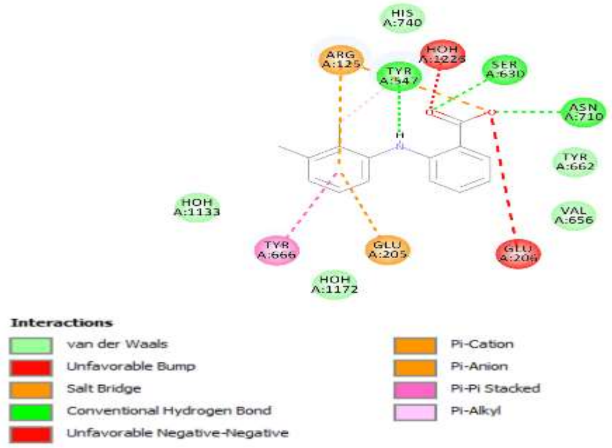

(b) Ketoprofen

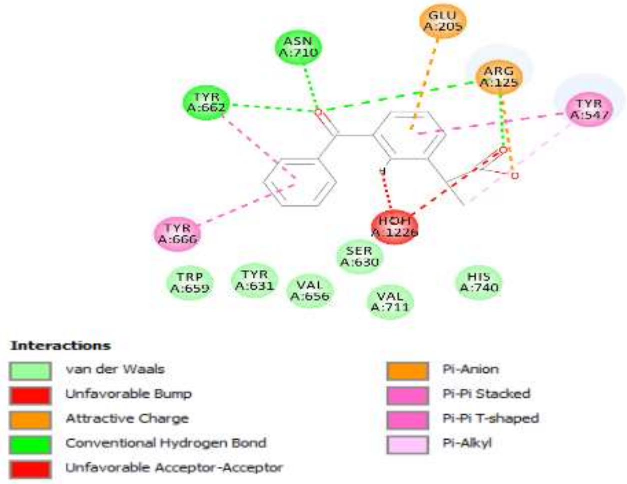

(c) Meloxicam

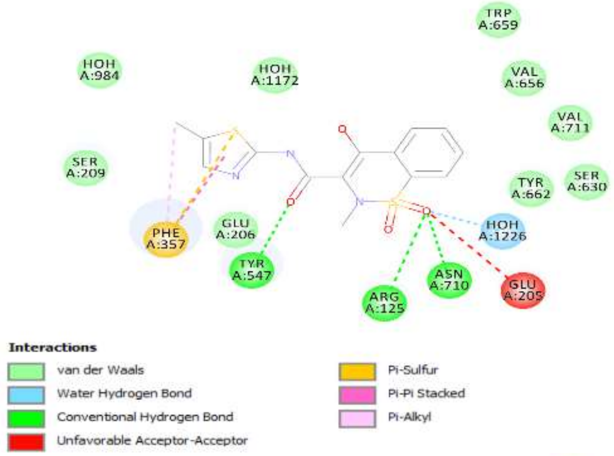

(d) Tolmetin

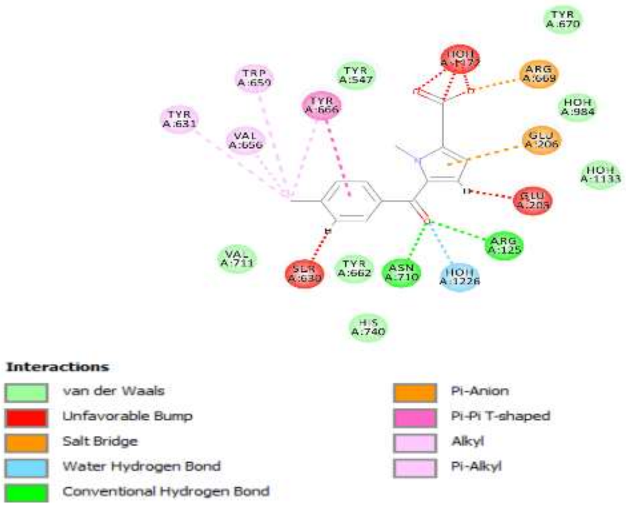

Figure S1. Two-dimensional ligand interactions with DPP-4 protein (PDB ID:6B1E). Non-bonded are depicted with different colors, and Discovery Studio Visualizer was used for the visualization of interactions.
